# Relationship of Obstructive Sleep Apnoea with Diabetic Retinopathy: A Meta-Analysis

**DOI:** 10.1155/2017/4737064

**Published:** 2017-11-05

**Authors:** Zhenliu Zhu, Fengying Zhang, Yunxia Liu, Shuqin Yang, Chunting Li, Qingliang Niu, Jinming Niu

**Affiliations:** ^1^Ophthalmology Department, Weifang Traditional Chinese Hospital, 1055 Weizhou Road, Kuiwen District, Weifang 261000, China; ^2^Otorhinolaryngological Department, Weifang Traditional Chinese Hospital, 1055 Weizhou Road, Kuiwen District, Weifang 261000, China; ^3^Outpatient Operating Room, Weifang Traditional Chinese Hospital, 1055 Weizhou Road, Kuiwen District, Weifang 261000, China; ^4^Otorhinolaryngological Department, Weifang People's Hospital, 151 Guangwen Street, Kuiwen District, Weifang 261000, China; ^5^Yantai KangAi Eye Hospital, 26 Zhichu Road, Zhifu District, Yantai 264000, China; ^6^Imaging Department, Weifang Traditional Chinese Hospital, 1055 Weizhou Road, Kuiwen District, Weifang 261000, China

## Abstract

Until now, the relationship of obstructive sleep apnoea (OSA) with diabetic retinopathy (DR) was controversial. This meta-analysis was performed to obtain definitive conclusion on this topic. Relevant articles were searched on databases of Pubmed, Google Scholar, and Chinese National Knowledge Infrastructure (CNKI). The articles were selected according to inclusion and exclusion criteria. Odds ratio (OR) with 95% confidence interval (CI) was used to evaluate the relationship of OSA with risk of DR. *I*^2^ and *P* value were used to assess the presence of heterogeneity. *I*^2^ ≥ 50% or *P* < 0.05 indicated significant heterogeneity. Sensitivity analysis was performed to evaluate the robustness of pooled results. Begg's funnel plot and Egger's regression analysis were adopted to assess publication bias. 6 eligible studies were selected in the present meta-analysis. The pooled results indicated that OSA was significantly associated with increased risk of DR (OR = 2.01, 95% CI = 1.49–2.72). Subgroup analysis based on type of diabetes mellitus suggested that OSA was related to DR in both Type 1 and Type 2 diabetes mellitus. Sensitivity analysis demonstrated that pooled results were robust. No significant publication bias was observed (*P* = 0.128). The results indicate that OSA is related to increased risk of DR.

## 1. Introduction

Obstructive sleep apnoea (OSA) is a disease featured by hypopnoea and/or apnoea during sleep that is commonly brought about by airway arousals and obstruction [1]. It affects 2% of women and 4% of men [2]. Obesity is a common risk factor for OSA and neck circumference is tightly correlated with the severity of OSA [3]. It has been estimated that about 75% of individuals with BMI > 40 kg/m^2^ have OSA [4]. OSA patients usually show extensive daytime sleepiness, cardiovascular disease, mood changes, and cognitive dysfunction [5]. In addition, OSA patients are more likely to develop metabolic syndrome, insulin resistance, vascular disease, and diabetes mellitus [6–9]. Moderate to severe OSA has been demonstrated to be related to enhanced risk of diabetes mellitus [10].

In addition, episodes of hypoxemia in OSA may be correlated with increased inflammatory mediators [11]. For these patients with diabetes mellitus, the levels of inflammatory mediators are also increased [12], which is significantly associated with diabetic microvascular complications, such as diabetic maculopathy and diabetic retinopathy (DR) [13]. It has been demonstrated that OSA in the patients with diabetic mellitus may be related with much more advanced diabetic maculopathy and more DR cases, which suggests the significant relationship of OSA with DR [14, 15].

Although extensive attention has been paid to the relationship of OSA with DR, no consistent conclusion has been obtained. The present meta-analysis was performed to extract a definitive conclusion on this issue. The outcome may contribute to screening OSA patients with high risk of DR and providing preventive strategy for these individuals.

## 2. Materials and Methods

### 2.1. Search Strategy

F. Zhang and Z. Zhu were responsible for searching articles on databases of Pubmed, Google Scholar, and Chinese National Knowledge Infrastructure (CNKI). The date was limited to June 2017. The terms used in the search were as follows: obstructive sleep apnoea OR OSA; diabetic retinopathy OR DR. Only the articles in English and Chinese were considered. We usually selected the recently published articles when we found two or more articles with same population. Meanwhile, we reviewed the references of obtained articles for relevant articles.

### 2.2. Inclusion and Exclusion Criteria

The articles were selected according to the inclusion and exclusion criteria. The inclusion criteria were listed as follows: (a) case-control studies; (b) the articles investigating the relationship of OSA and DR; (c) the articles providing the frequency of DR in case (OSA+) and control (OSA−) groups. Review articles and case reports would be excluded from the analysis.

### 2.3. Data Extraction

Data extraction was independently performed by two authors (Y. Liu and S. Yang). The controversial issues would be solved with a discussion with the third author (F. Zhang). They usually reviewed the data together and had discussion until consistent opinion was reached. The data were name of first author, year of publication, country, sample size, type of diabetes mellitus, and frequency of DR in each group.

### 2.4. Statistical Analysis

All the analysis was performed in Stata 12.0 software. Odds ratio (OR) with corresponding 95% confidence interval (CI) was calculated to evaluate the relationship of OSA with DR. *I*^2^ and *P* value were used to assess the existence of heterogeneity. *I*^2^ ≥ 50% or* P* < 0.05 indicated significant heterogeneity. The data was pooled with the random-effects model if the heterogeneity presented; otherwise, it was pooled with the fixed-effects model. Sensitivity analysis was performed to evaluate the robustness of pooled outcomes. Begg's funnel plot and Egger's regression analysis were adopted to detect potential publication bias.

## 3. Results

### 3.1. Article Selection Process

**Figure 1:**
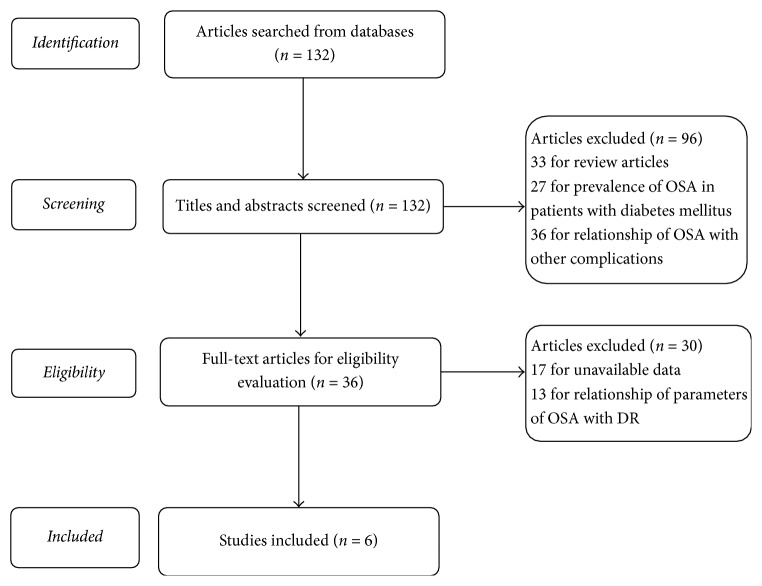
Selection process of eligible articles. 6 eligible articles were selected.

After rough search, a total of 132 relevant articles were obtained. Then the titles and abstracts were screened. Subsequently, 96 articles were removed for review articles (*n* = 33), investigating prevalence of OSA in patients with diabetes mellitus (*n* = 27) and analyzing the relationship of OSA with other complications of diabetes mellitus (*n* = 36). For the remaining 36 articles, 30 articles were removed for unavailable data (*n* = 17) and analyzing the relationship of parameters of OSA with DR (*n* = 13). At last, 6 eligible studies were included [14, 16–20]. The selection process was shown in Figure 1. The detailed information of each study was listed in Table 1.

### 3.2. Relationship of Obstructive Sleep Apnoea with Diabetic Retinopathy

**Figure 2:**
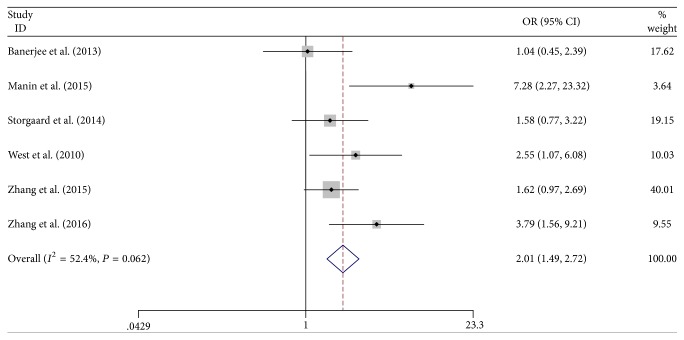
Relationship of OSA with DR. The results indicated that OSA was significantly associated with risk of DR (OR = 2.01, 95% CI = 1.49–2.72).

The outcome about the relationship of OSA with DR was shown in Figure 2. The pooled data was calculated with the fixed-effects model. It suggested that OSA was significantly related to increased risk of DR (OR = 2.01, 95% CI = 1.49–2.72). Subgroup analysis by type of diabetes mellitus was also performed. OSA was associated with the risk of DR among both of patients with Type 1 and Type 2 diabetes mellitus.

### 3.3. Sensitivity Analysis and Publication Bias Detection

**Figure 3:**
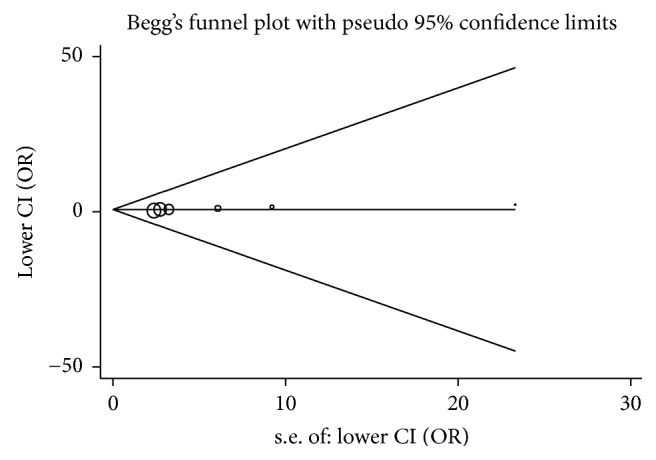
Begg's funnel plot. No significant publication bias was observed (*P* = 0.128).

Sensitivity analysis was performed to assess the robustness of pooled results. It demonstrated that the pooled results were robust. Publication bias was detected via Begg's funnel plot (Figure 3) and Egger's regression analysis. In the present meta-analysis, no obvious publication bias was observed (*P* = 0.128).

## 4. Discussion

The present meta-analysis suggested that OSA was significantly associated with increased risk of DR. The outcome was observed in both Type 1 and Type 2 diabetes mellitus. In the analysis, no obvious heterogeneity and publication bias were observed. The pooled results were credible.

The association between OSA and Type 2 diabetes mellitus has been well defined. Onset of OSA is correlated with increased insulin resistance/glucose intolerance. Meanwhile, diabetes mellitus may aggravate sleep-disordered breathing due to autonomic neuropathy [21]. It is also reported that insulin resistance could predict the onset of OSA. Therefore, the association between insulin resistance and OSA seems to be bidirectional [22]. In addition, Type 2 diabetes mellitus shares same risk factor with OSA, such as obesity [23, 24]. Obesity could cause insulin resistance/glucose intolerance. Meanwhile, it appears as an important risk factor of OSA. It has been estimated that prevalence of OSA ranges from 17% to 86% in patients with Type 2 diabetes mellitus in the Japan, US, and Hong Kong [25–27].

DR is one of microvascular complications of diabetes mellitus, which presents as a main cause of visual impairment among working-age population. Thus, early diagnosis of DR in the patients with diabetes mellitus is vital. However, the pathogenesis of DR is not fully explained up to now [28, 29]. Known risk factors of DR include older age, longer duration of diabetes mellitus, cardiovascular events, and aging [30, 31]. Alcohol intake is also regarded as one risk factor of DR [32]. The recent studies found significant relationship of OSA with risk of DR.

About the relationship of OSA with DR, there was no consistent conclusion until now. On this topic, Leong et al. also initiated a meta-analysis. Their analyses suggested that minimum oxygen saturation showed impact on DR (OR = 0.91, 95% CI = 0.87–0.95) [33]. The cross-sectional study by Manin et al. reported that OSA was correlated with the development of macrovascular complications and retinopathy [17]. In another study by West et al., retinopathy and maculopathy scores were found to be much worse in the OSA group. Multiple regression analysis suggested that OSA was an independent predictor of retinopathy (*R*^2^ = 0.19, *P* < 0.0001) [14]. Banerjee et al. investigated the potential relationship of OSA and DR among patients with severe obesity and concluded that there was no significant relationship of OSA and diabetic retinal complications [16].

Our analysis was based on 6 eligible articles and the results were credible. However, the sample size was relatively small, which may affect the accuracy of results. Moreover, much larger-scale research, such as multiple-centre study, should be performed to confirm the results. In addition, the pathogenesis of DR involves many factors; only OSA was analyzed in the present meta-analysis, which cannot fully explain the developmental mechanism of DR. More comprehensive analysis will contribute to uncovering the pathogenesis of DR and more factors should be considered in the future analysis. Meanwhile, most studies analyzed Type 2 diabetes mellitus and only one study involved Type 1 diabetes mellitus. The relationship of OSA with DR in patients with Type 1 diabetes mellitus should be verified.

## 5. Conclusion

In conclusion, the presence of OSA is related to increased risk of DR. The conclusion is helpful for screening the individuals with high risk of DR among the patients with diabetes mellitus. It is significant for clinical physicians.

## Figures and Tables

**Table 1:** Main information about the included studies.

Author	Year	Country	Sample size, *n*		Type of DM
			OSA+	OSA−	
Banerjee	2013	United Kingdom	46	47	Type 2
Manin	2014	France	31	36	Type 1
Storgaard	2014	Denmark	72	108	Type 2
West	2010	United Kingdom	28	90	Type 2
Zhang	2015	China	310	162	Type 2
Zhang	2016	China	121	41	Type 2
